# The Effectiveness of Telerehabilitation as a Supplement to Rehabilitation in Patients After Total Knee or Hip Replacement: Randomized Controlled Trial

**DOI:** 10.2196/14236

**Published:** 2019-11-07

**Authors:** Sarah Eichler, Annett Salzwedel, Sophie Rabe, Steffen Mueller, Frank Mayer, Monique Wochatz, Miralem Hadzic, Michael John, Karl Wegscheider, Heinz Völler

**Affiliations:** 1 Center of Rehabilitation Research University of Potsdam Potsdam Germany; 2 Department of Computer Science and Therapy Science Trier University of Applied Science Trier Germany; 3 University Outpatient Clinic Sports Medicine and Sports Orthopedics University of Potsdam Potsdam Germany; 4 Fraunhofer Institute for Open Communication Systems Berlin Germany; 5 Institute of Medical Biometry and Epidemiology University Medical Center Hamburg-Eppendorf Hamburg Germany

**Keywords:** telerehabilitation, home-based, total hip replacement, total knee replacement, exercise therapy, aftercare, rehabilitation

## Abstract

**Background:**

Telerehabilitation can contribute to the maintenance of successful rehabilitation regardless of location and time. The aim of this study was to investigate a specific three-month interactive telerehabilitation routine regarding its effectiveness in assisting patients with physical functionality and with returning to work compared to typical aftercare.

**Objective:**

The aim of the study was to investigate a specific three-month interactive telerehabilitation with regard to effectiveness in functioning and return to work compared to usual aftercare.

**Methods:**

From August 2016 to December 2017, 111 patients (mean 54.9 years old; SD 6.8; 54.3% female) with hip or knee replacement were enrolled in the randomized controlled trial. At discharge from inpatient rehabilitation and after three months, their distance in the 6-minute walk test was assessed as the primary endpoint. Other functional parameters, including health related quality of life, pain, and time to return to work, were secondary endpoints.

**Results:**

Patients in the intervention group performed telerehabilitation for an average of 55.0 minutes (SD 9.2) per week. Adherence was high, at over 75%, until the 7th week of the three-month intervention phase. Almost all the patients and therapists used the communication options. Both the intervention group (average difference 88.3 m; SD 57.7; *P*=.95) and the control group (average difference 79.6 m; SD 48.7; *P*=.95) increased their distance in the 6-minute-walk-test. Improvements in other functional parameters, as well as in quality of life and pain, were achieved in both groups. The higher proportion of working patients in the intervention group (64.6%; *P*=.01) versus the control group (46.2%) is of note.

**Conclusions:**

The effect of the investigated telerehabilitation therapy in patients following knee or hip replacement was equivalent to the usual aftercare in terms of functional testing, quality of life, and pain. Since a significantly higher return-to-work rate could be achieved, this therapy might be a promising supplement to established aftercare.

**Trial Registration:**

German Clinical Trials Register DRKS00010009; https://www.drks.de/drks_web/navigate.do? navigationId=trial.HTML&TRIAL_ID=DRKS00010009

## Introduction

### Background

According to data from the Organization for Economic Cooperation and Development (OECD), 299 total hip and 206 total knee replacements were performed per 100,000 people in Germany in 2015. With these numbers, Germany ranks second (hip) and fourth (knee) in the world [1]. A further increase in endoprosthetic interventions on the knee and hip joint is to be expected due to an aging society and an increasing rate of obesity [2-4].

After an orthopedic procedure, rehabilitation as a multidisciplinary approach can improve the function of the joints and the ability to maintain a normal daily life, as well as relieve a patient’s pain [5-7]. The effectiveness of rehabilitation after a knee or hip replacement has already been documented with substantial evidence [8-12]; however, maintaining the achieved therapeutic outcome remains a challenge. Prior studies have reported various barriers to using rehabilitation services, such as miscellaneous financial, structural, personal, and attitudinal determinants of access to rehabilitation [13]. Currently, there is an ongoing study, whose results will soon be published, [14] that seeks to determine barriers to using rehabilitation services.

In Germany, patients are offered numerous aftercare options, such as the multimodal intensified program (IRENA) or training rehabilitation aftercare (T‑RENA), but only about half of eligible patients take advantage of them [15]. Therefore, to improve the sustainability of postoperative therapies, more flexible and individualized offers need to be developed.

In this regard, telerehabilitation seems to be the obvious choice since it can be performed irrespective of location and time, and it has the potential to increase both utilization and therapy adherence. The current telerehabilitation offerings should be adapted to the individual and indication‑specific needs of the patients and should enable contact with the supervising therapists. However, this could not be investigated with the currently available systems, as they are either not specific enough for the indications of a patient or do not offer a tool to communicate with a therapist [16-22]. The telerehabilitation systems studied until now often differ in terms of their communication structures and their feedback options. Thus, Moffet et al [17] and Tousignant et al [19] investigated synchronous telerehabilitation applications where the physiotherapist and the patient communicated in real-time via videoconferencing, while Bini et al [16] and Piqueras et al [18] studied systems in which the communication between therapist and patient took place with a time delay. Additionally, the latter group of papers used a system with sensor-based kinematic feedback on motion execution. It is well known that the addition of external feedback can contribute to improved movement performance [23]. Regarding kinematic feedback systems, prior investigations on motion detection using a Kinect sensor already exist. In two studies, the acceptable reliability and validity of a Kinect-based motion analysis was demonstrated when compared to other marker-based kinematic measurement systems [24,25]. Both authors concluded that the Kinect may be a suitable tool for analyzing movement in the clinical field.

Hence, the MeineReha system [26,27] combines many components that were thus far investigated individually and additionally provides real-time visual feedback using a Kinect camera. Following development and validation [28], the MeineReha system was supplemented with an individualized and therapist‑controlled telerehabilitation program consisting of 38 training exercises available for patients after their knee and hip replacements.

### Aim of the study

The aim of this randomized controlled trial was to examine previously developed telerehabilitation therapy in terms of its functional parameters, quality of life, and pain relief, as well as time to return to work, compared to usual aftercare programs.

## Methods

### Patients

**Figure 1:**
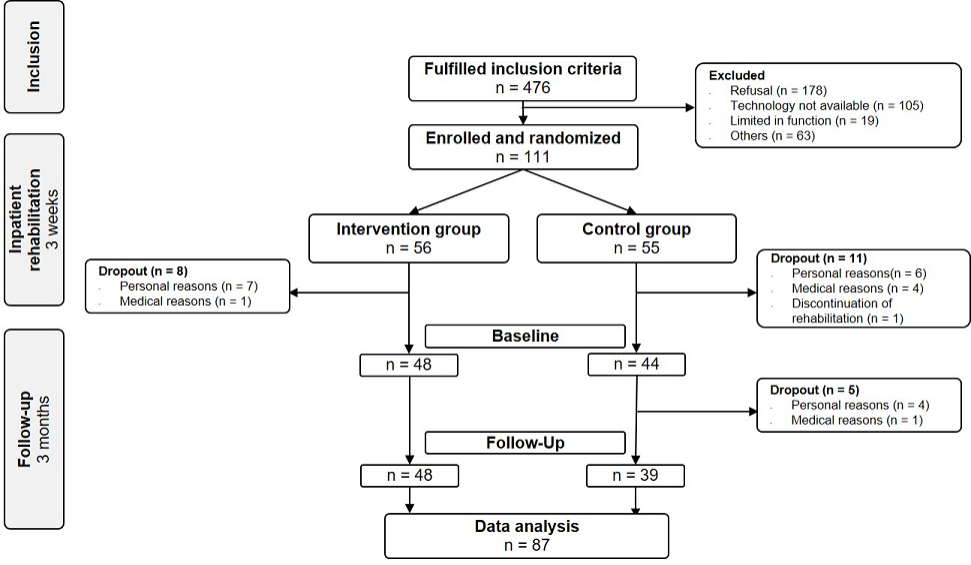
Consolidated Standards of Reporting Trials flow chart for the inclusion process.

From August 2016 to December 2017, after a screening of 476 patients in three inpatient rehabilitation centers, 111 patients were included in the randomized controlled trial (Figure 1). Patients were eligible for inclusion if a total hip or knee replacement was performed following idiopathic, posttraumatic, or congenital osteoarthritis, if they were aged between 18-65 years, and if they were insured by the national or regional German Pension Insurance. Patients not expected to achieve functional safety in walking with full load by the end of the rehabilitation were excluded. For those patients, it was assumed that they would not be able to perform exercises with adequate load or the assessments at the study site. Insufficient verbal and written German-language skills also led to exclusion. For the use of the telerehabilitation system at home, some additional criteria (eg, High Definition Multimedia Interface [HDMI]-compatible screen, minimum 2.5-meter space in front of the screen, and internet access) were required for the patients at home. After enrollment, patients were assigned to either the intervention group (IG) or the control group (CG) using block randomization in a 1:1 ratio and based on randomization lists drawn up in advance by the biometric institute. Written consent was obtained from all patients.

### Intervention

**Figure 2:**
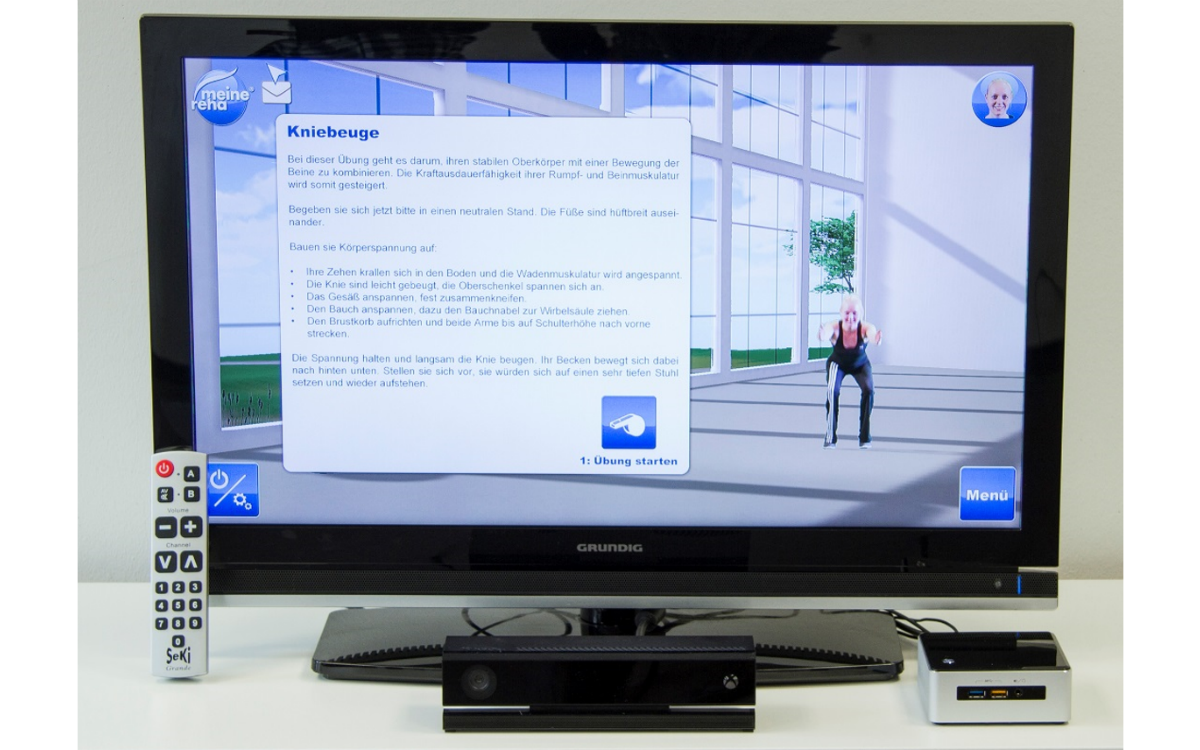
Hardware of the telerehabilitation system and an example exercise demonstrated by a virtual avatar.

Following three weeks of inpatient rehabilitation, the patients assigned to the IG performed a three-month, home-based telerehabilitation program based on the MeineReha system, which consisted of a home component as well as a working portal for the therapist in the clinic. The main component, from the patient's perspective, was the MeineReha application that was installed on the rehab box at home. The rehab box (minicomputer with internet access) was connected to the usual peripherals (mouse and keyboard) as well as to a screen and Kinect sensor (camera) (Figure 2).

The exercises to build up strength and improve postural control were chosen by the supervising therapist from a previously developed exercise catalog. The training intensity was individualized in terms of the choice of exercises, the number of sets and repetitions, and the duration of the breaks, which could all be adjusted by the therapist. Patients were asked to perform the training three times a week. There were different options for the patient and the therapist to communicate with each other: (1) the patient could record and send audio messages to their therapist whenever they wanted and the therapist was able to listen to it whenever their schedule gave them time to do it; (2) the therapist could respond or start a conversation with their patient at any time with individualized text messages, which the patient was shown whenever they started the system (eg, therapists could either remind the patient to do their exercises more often or just ask them about their condition); and (3) the patient and the therapist were able to make appointments for live video conferences, which they were supposed to conduct on a weekly basis to perform individual training consultation or to allow for the patient to ask questions about their training.

**Figure 3:**
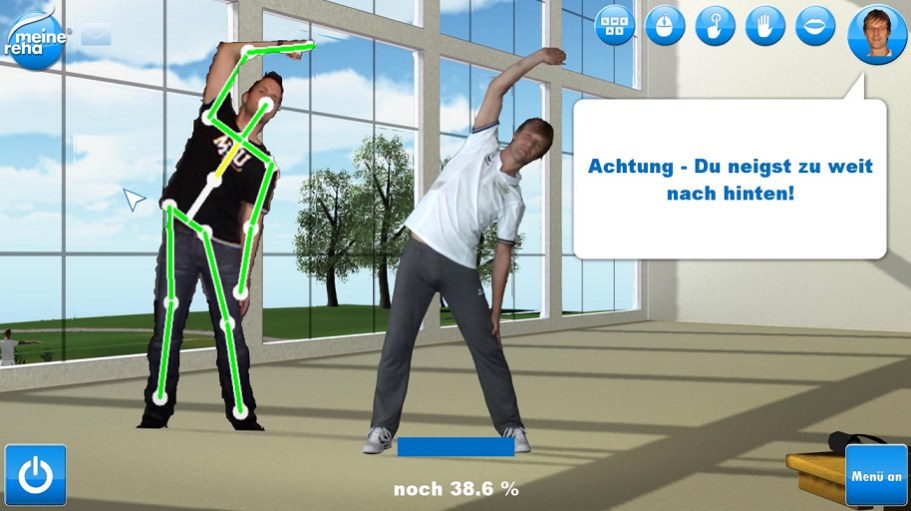
Real-time feedback during an exercise.

During the training, the exercises were demonstrated on screen by an avatar (Figure 3). The patient performed the exercises simultaneously and was detected by means of a Kinect sensor (camera). The system compensated for a patient’s movement patterns with a predetermined target movement and sent them real‑time visual feedback in which their relevant body segments were colored green for correct movements and red in the case of incorrect movements (Figure 3). The quality of each exercise was demonstrated to the patient following the performance of the exercise by using a school grade and the percentage of red and green values. The grading algorithm considered the synchronicity of the movements, the compliance with the target movement, and the number of repetitions. For training supervision, the therapist was given access to the frequency of the training as well as the exercise evaluations.

### Control Group

Patients in the control group did not receive any study-specific therapy after their inpatient rehabilitation. The follow-up was carried out identically to the IG three months after randomization. The patients of both groups were also offered the usual aftercare, that is IRENA and physiotherapy.

### Data Collection

To verify the patients’ adherence, the process data for the IG (ie, frequency and duration of training, use of communication options) were read from the system. In addition, the frequency and duration of training and the use of other aftercare therapies were recorded by all patients in their training diaries. Further, all patients were investigated for functional parameters (eg, the 6-minute walk test, the Stair Ascend test, the Five-Times-Chair-Rise test, and the Timed-Up-and-Go test) at the study site (University of Potsdam) after the inpatient rehabilitation. Further, subjective parameters such as the Short Form Health Survey-36 (SF-36) on health-related quality of life, pain on the operated joint, stiffness, and function were assessed using the Western Ontario and McMaster Universities Arthritis Index (WOMAC), with a patient’s ability to return to work also being assessed. In addition, patient characteristics, comorbidities, and medications were documented. The investigations were repeated after three-months follow-up. In terms of acceptance, we collected data from the IG using the Telehealth Usability Questionnaire (TUQ), including the concepts ease of use, learnability, satisfaction, future use, and reliability, on a 7-point Likert scale, with a 1 meaning disagree and a 7 meaning agree. To achieve comparability of the scales, we normalized the results as a quotient of the sum of the raw values and the total number of items, multiplied by a factor of 100.

### Statistical Analyses

The statistical analyses were conducted according to the description in the previously published study protocol [29]. All analyses were performed with the full analysis set of randomized patients (modified intention-to-treat). Patient characteristics and follow-up values ​​were described with mean and standard deviation (metric variables), and absolute and relative frequencies (categorical variables). Group-specific changes in metric variables (trends) were tested for significance versus no change with one-factorial variance analyses. The calculation of the number of cases (n=84) was based on the comparison of the primary endpoint (improvement in the 6-minute walk test) between the groups. This comparison was carried out with an analysis of covariance (ANCOVA) with 22 baseline covariates at the 5% level (two-sided). All metric secondary endpoints were tested analogously without multiple adjustment. The ANCOVA estimates of the group differences in the continuous endpoints are presented in a forest plot. The group difference in the return-to-work rates was tested with the Chi-squared test. At the end, an analysis was performed within the IG on whether the improvement in the 6-minute walk test was dependent of the number of training units, the number of text messages sent by the therapist, or the number of audio messages sent by the patient.

## Results

### Patient Characteristics

At baseline, data from 87 patients from the IG (n=48) and the CG (n=37) (Figure 1) could be analyzed. The patients were an average of 54.9 (SD 6.7) years old, with an average of 56.8 (SD 5.7) for the CG and an average of 53.3 (SD 7.0) years (*P*=.012) for the IG. Overall, 51.7% (45/87) were female and had an above-average level of education (43.7% with a polytechnic or university degree). About two-thirds (69.0%; 60/87) of the patients received hip replacements, 31.0% (27/87) knee replacements, almost half of the patients (43.7%; 38/87) were obese (body mass index [BMI]≥30 Kg/m²), a third (36.8%; 32/87) of the patients had a cardiac comorbidity, and about a quarter (24.1%; 21/87) of the patients had an orthopedic comorbidity. At baseline, 48.4 days (SD 13.1) after surgery, one in ten patients (9.2%; 8/87) was treated with opioids and half of the patients (49.4%; 43/87) with nonopioid analgesics. Before elective surgery, 87.4% (76/87) of patients were gainfully employed. Table 1 shows the corresponding baseline figures of 87 patients with functional parameters at follow-up, which were eligible for the multivariate analysis. Categorical variables are expressed as absolute and relative frequencies with n (%), and metric variables as mean (SD).

**Figure 4:**
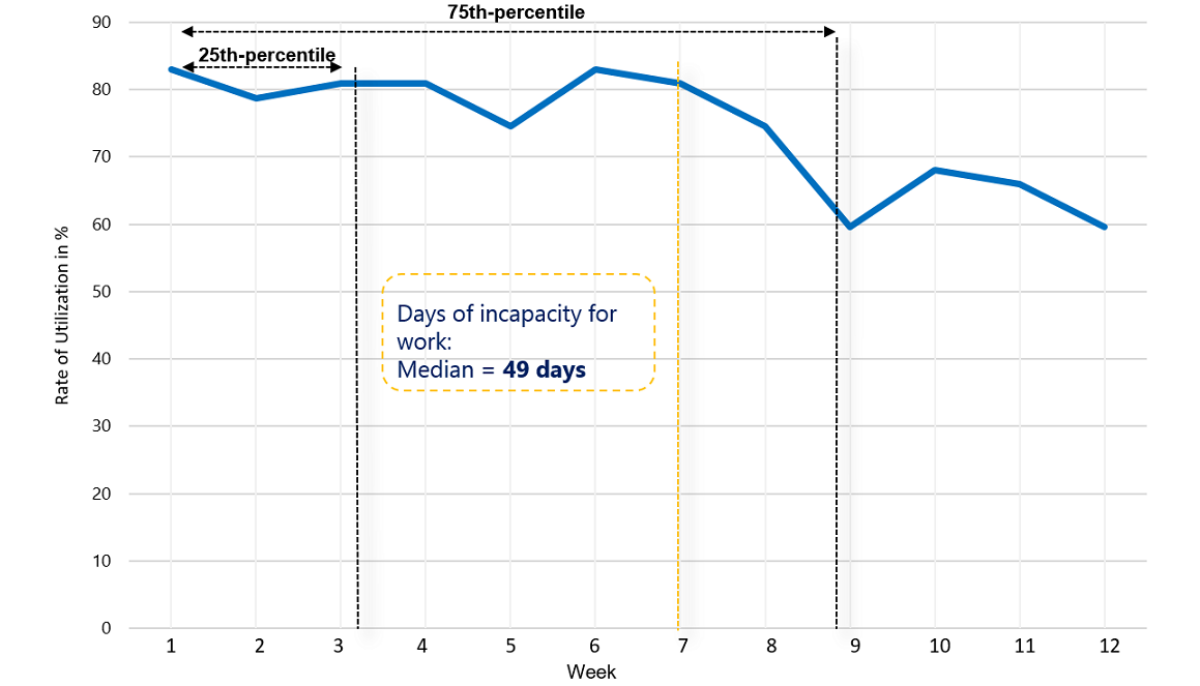
Utilization of telemedical assisted exercise therapy.

According to the self-reported exercise diary, the patients in the IG performed their telerehabilitation an average of 55.0 minutes (SD 9.2) per week. The data read from the system showed a training duration of 39.0 minutes (SD 8.0). The participation rate was over 75% until the 7th week of the three-month intervention phase, but afterwards it decreased in parallel to the return to work (Figure 4). More than half of the patients continued their telerehabilitation after the 7th week until the end of the 12-week intervention. The communication via text and voice messages between the patients and therapists was used during the first few weeks. At the beginning of the intervention phase, almost all of the patients and therapists contacted each other (98% of the therapists sent text messages and 88% of the patients sent voice messages), but after the 4th week there was only a little communication (<50% sent messages). Overall, the patients sent an average of 6.0 audio messages (SD 5.9), while the therapists sent a mean of 7.0 text messages (SD 4.5) during the 12-week intervention. Furthermore, patients in both the IG and CG used regular rehabilitation aftercare. A total of 51.3% (20/39) of the CG and 33.3% (16/48) of the IG used IRENA aftercare, and physiotherapy was conducted by 81.3% (39/48) of the IG and 71.8% (28/39) of the CG.

**Table 1:** Patient characteristics (n=87).

Characteristics			Control group (n=39)	Intervention group (n=48)	Total cohort (n=87)	*P* value
**Socio-demographic data, lifestyle, and postoperative period**						
	Age (years), mean (SD)		56.8 (5.7)	53.3 (7.0)	54.9 (6.7)	.012
	Sex (female), n (%)		19 (48.7)	26 (54.2)	45 (51.7)	.61
	**BMI^a^ (kg/m²), mean (SD)**		**30.3 (4.9)**	**29.8 (5.9)**	**30.0 (5.5)**	**.52**
		Normal weight: 18.5–<25, n (%)	5 (12.8)	8 (16.7)	13 (14.9)	.86
		Overweight: 25–<30, n (%)	17 (43.6)	19 (39.6)	36 (41.4)	—^b^
		Obesity: ≥30, n (%)	17 (43.6)	21 (43.8)	38 (43.7)	—
	Smoking behavior (smoker), n (%)		10 (25.6)	13 (27.1)	23 (26.4)	.88
	Time from surgery to admission of inpatient rehabilitation (days), mean (SD)		18.4 (8.8)	18.8 (12.9)	18.6 (11.3)	.61
	Time of inpatient rehabilitation (days), mean (SD)		23.3 (3.7)	23.3 (3.5)	23.3 (3.5)	.77
	Time from surgery to baseline investigation (days), mean (SD)		50.4 (11.6)	46.9 (14.1)	48.4 (13.1)	.11
**Education and occupation**						
	**Graduation, n (%)**					**.82**
		Less than general or subject-linked higher education entrance qualification	21 (53.8)	27 (56.3)	48 (55.2)	
		General or subject-linked higher education entrance qualification	18 (46.2)	21 (43.8)	39 (44.8)	
	**Vocational education, n (%)**					**.20**
		Less than polytechnic or university degree	19 (48.7)	30 (62.5)	49 (56.3)	
		Polytechnic or university degree	20 (51.3)	18 (37.5)	38 (43.7)	
	Gainfully employed, n (%)		34 (87.2)	42 (87.5)	76 (87.4)	.96
	Unemployed, n (%)		2 (5.1)	2 (4.2)	4 (4.6)	.83
	Incapacity for work before surgery (days), mean (SD)		16.6 (49.9)	21.3 (47.3)	19.2 (48.2)	.27
	Work intensity (moderate/severe), n (%)		8 (20.5)	9 (18.8)	17 (19.5)	.84

^a^BMI: body mass index

^b^Not applicable

### Functional Parameters

**Figure 5:**
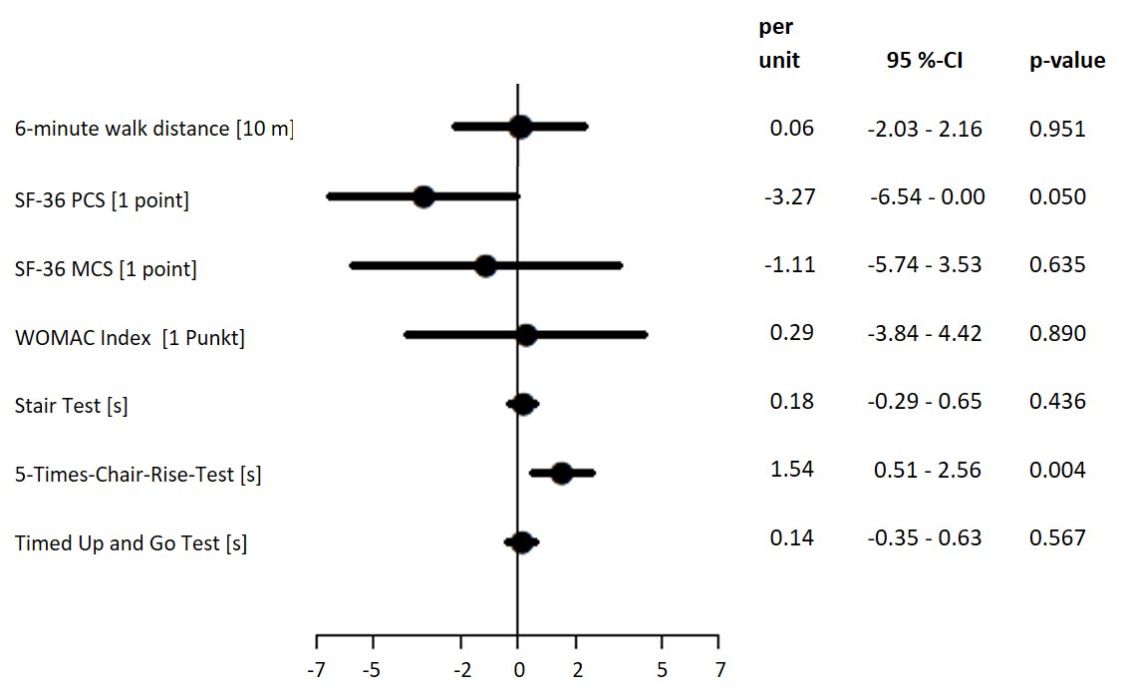
Differences in endpoints between intervention and control group, multiple adjusted. SF: Short Form Health Survey; PCS: physical component scale; MCS: mental component scale; WOMAC: Western Ontario and McMaster Universities Osteoarthritis Index.

The patients in the IG could increase their 6-minute walking distance from an average of 440.6 (SD 78.2) to 530.4 meters (SD 79.0) (Difference [Delta]=88.3 m; SD 57.7 m; *P*<.001), and the patients in the CG from 433.3 (SD 80.2) to 513.0 meters (SD 70.6) (Delta=79.6 m; SD 48.7; *P*<.001) (Table 2). In the multivariate analysis, no group difference could be detected (*P*=.95) (Figure 5). The improvement within the intervention group was associated with the number of audio messages sent by the patient (*P*=.02), but not with the number of text messages sent by the therapist (*P*=.49) or with the number of training units (*P*=.07).

Other functional parameters (eg, the Timed Up and Go Test, the Stair Ascend Test, and the Five Times Chair Rise Test) also showed similar improvements in both groups (Table 2). The only multivariate significant group difference could be shown in the Five Times Chair Rise Test (*P*=.004) (Figure 3).

**Table 2:** Functional und subjective parameters (n=87). All values presented as mean (SD).

Parameter	Baseline			Follow-up			Differences (Delta)	
	IG^a^	CG^b^	*P* value	IG	CG	*P* value	IG	CG
6-minute walk test (m)	440.6 (78.2)	433.3 (80.2)	.90	530.4 (79.0)	513.0 (70.6)	.43	88.3 (57.7)	79.6 (48.7)
Stair Ascend Test (s)	8.7 (2.7)	8.6 (4.0)	.33	6.2 (1.2)	6.1 (1.5)	.44	–2.5 (2.4)	–2.5 (3.0)
Timed Up and Go Test (s)	9.3 (1.8)	9.0 (2.4)	.16	7.5 (1.2)	7.5 (1.6)	.93	–1.9 (1.5)	–1.5 (2.2)
Five Times Chair Rise Test (s)	16.9 (3.7)	17.1 (6.2)	.38	14.2 (2.7)	13.2 (2.3)	.06	–2.7 (3.5)	–3.8 (5.1)
SF-36^c^ PCS^d^	33.8 (7.6)	33.3 (7.9)	.82	44.6 (9.9)	44.4 (8.3)	.80	10.7 (10.4)	11.1 (7.2)
SF-36 MCS^e^	54.8 (10.6)	53.9 (11.8)	.98	52.4 (10.6)	54.1 (9.8)	.28	–2.5 (12.4)	0.1 (8.5)
WOMAC^f^ Index	26.4 (18.5)	24.8 (16.4)	.78	11.5 (12.7)	13.9 (14.3)	.51	–14.9 (13.6)	–10.9 (13.5)

^a^IG: intervention group

^b^CG: control group

^c^SF-36: Short Form Health Survey-36

^d^PCS: physical component scale

^e^MCS: mental component scale

^f^WOMAC: Western Ontario and McMaster Universities Osteoarthritis Index

### Health-Related Quality of Life, Pain, and Return to Work

Regarding the health-related quality of life on the SF-36, an improvement in the physical component scale (PCS) was achieved in both groups. Furthermore, the WOMAC Index showed a significant reduction in both groups (Table 2). At the end of the intervention, 31 patients (64.6%) from the IG and 18 patients (46.2%) from the CG were gainfully employed (*P*=.01).

### Acceptance of the Telerehabilitation System

In terms of acceptance of the TUQ, the patients of the IG showed high consent in the normalized values of the scales of ease of use and learnability (mean 85.2; SD 2.9) as well as in satisfaction and future use (mean 79.8; SD 3.2), whereas the values of the reliability scale were lower (mean 51.8; SD 3.7).

## Discussion

### Summary

In short, the use of telerehabilitation with patients having just undergone knee or hip replacements was equivalent to the usual aftercare in terms of the difference achieved in the 6-minute walk test. In addition, equivalent increases in both groups were demonstrated as secondary endpoints for functional mobility, health-related quality of life, and joint-related complaints. However, the patients in the intervention group were employed at a significantly higher rate at the end of the intervention.

The patients in the intervention group intensively used the telerehabilitation as a complementary aftercare option for a prolonged period of the study. The difference between the training durations given by the patient and those read out of the system can be explained by the preparation and cool-down times of the exercises, since only the exact execution time of the exercises was measured in the system. Likewise, the communication possibilities of the system, in terms of using text and voice messages, were exhausted. It is self-evident that the need for close contact with the therapist diminishes over a longer period, because the patients eventually either returned to work or all their questions about the system and training had already been answered.

The usual aftercare treatment was also used extensively by the patients in both groups. The participation rate of the control group (51%) in the IRENA aftercare program was comparable to the participation rate (50%) for the medical rehabilitation aftercare (MERENA) program in patients with chronic back pain [15]. More than 30% of the IG patients also used IRENA. About 80% of the IG performed their telerehabilitation until the 7th week of the three‑month intervention phase, and even in the following weeks participation rates of more than 60% could be achieved. The high rate of use of the telerehabilitation may be due to its being time- and place-independent, as occupational obligations are a major obstacle to participation in aftercare [15]. Our study results further show that even after a sizeable proportion of the patients returned to work there was still good adherence to the telerehabilitation. This indicates the practicability of this program for patients of working age following a knee and hip replacement. Thus, in another study, good adherence to telerehabilitation for patients having undergone knee replacements could also be researched [17].

During the three-month investigation phase, a significant increase in the 6-minute walking distance was recorded in both groups. For the population of knee and hip replacement patients, an improvement of 50-60 meters in the 6-minute walk test is considered clinically relevant [30]. The values at follow-up showed only small deviations from the normal values for healthy individuals, with 578 meters for men and 534 meters for women in the 50-60 years old age group [31], which shows that patients' functionality seemed to be largely restored four to five months post operation. Furthermore, the baseline values of the patients had already exceeded those of comparable clinical populations [30,32,33] and thus indicated a high initial level of physical performance. It is possible that patients had already improved their functionality in the inpatient rehabilitation to such a high extent that there was little potential for further improvement.

Consistent with the results of the 6-minute walking test, further functional mobility tests with significant improvements in both groups demonstrated the equivalence of the telerehabilitation. In the Five Times Chair Rise Test, the control group had a statistically significant and higher improvement, however, the difference between the groups was significantly below the clinically relevant value of 2.5 seconds [34], which relativizes the group difference despite its significance.

As for the WOMAC Index, values below 29.5 points are considered a treatment success for patients after a knee replacement [35]. The score achieved by the patients at baseline was already below this cut-off value. Furthermore, this value decreased significantly during the intervention phase in both groups and was below the postoperative WOMAC scores of comparable clinical populations [36-39].

For health-related quality of life, both groups achieved a significant increase on the physical component scale during the study phase. Against the background of the mainly physically oriented aftercare programs, this enhancement seems reasonable. However, despite the improvement, at the end of the intervention patients were slightly below the age‑related normative values of 47-49 points, with an average of 44 points [40]. For the intervention period of three months, similar values can be found for patient populations after knee and hip replacement [32,33]. The results of the mental component scale did not change significantly for either group but were slightly above the norm of 48-50 points at the end of the intervention [40].

Although most of the investigated endpoints did not show the superiority of the telerehabilitation, a significantly higher proportion of the IG returned to work at the end of the three-month study period. However, this fact cannot be explained by improved physical performance, quality of life, or reduced joint‑related complaints of the intervention group. It remains to be discussed whether the possibility of performing telerehabilitation regardless of time and place could have led to an earlier return to work by the IG. In addition, the high dropout rate of the control group (29.1%; 11/39) compared to the intervention group (14.1%; 7/48) should also be considered. Given the route to the study site, as well as the time of about two hours required for each baseline and follow-up investigation, there exists the possibility that the CG patients who returned to work were no longer willing to participate in the study.

### Limitations

In the investigated sample, an above-average education level can be ascertained (43.5% with a polytechnic or university degree). Data from the Employment Agency in Germany shows that, in the total population, only 20% of gainfully employed individuals have a polytechnic or university degree [41]. Furthermore, the 5.4% unemployment rate of the sample should be classified as low compared to the 8.4% Berlin average [42]. In addition, a substantial proportion of patients came from Berlin and the surrounding countryside. Therefore, in this study, the access route to the study site that the patients had to traverse twice independently may have been an obstacle to the participation of patients from more distant, infrastructurally weak areas. Only a quarter of the screened patients participated in the study. Thus, the low participation rate and the discussed patient characteristics suggest a selection bias.

Another limitation of the study design is the lack of blinding of study participants and investigators. As a result, this is a possible influence on the participants during the investigations that cannot be excluded. It is known that nonblinded studies can demonstrate greater intervention effects than blinded ones [43].

All patients underwent inpatient rehabilitation and aftercare treatment. It is not possible to determine which improvements can be directly traced back to the effect of telerehabilitation, as due to ethical reasons the usual aftercare programs in this study were not replaced but instead complemented by the new approach.

### Conclusion

The investigated telerehabilitation for patients having undergone knee or hip replacement was equivalent to the usual aftercare treatments in terms of improvements in the 6-minute walk test and in other functional parameters. However, at the end of the intervention, patients in the intervention group returned to work at a significantly higher rate. These results suggest that the system is complementary to the established aftercare programs in Germany (eg, IRENA or T-RENA), especially in infrastructurally weak areas.

## Appendix

Multimedia Appendix 1 CONSORT-EHEALTH checklist (V 1.6.2).

## Abbreviations

ANCOVA: analysis of covariance
BMI: body mass index
CG: control group
HDMI: High Definition Multimedia Interface
IG: intervention group
IRENA: multimodal intensified aftercare
MERENA: medical rehabilitation aftercare
OECD: Organization for Economic Cooperation and Development
PCS: physical component scale
SF-36: Short Form Health Survey-36
T‑RENA: training rehabilitation aftercare
TUQ: Telehealth Usability Questionnaire
WOMAC: Western Ontario and McMaster Universities Arthritis Index

## References

[1] OECD (2017). Health at a Glance 2017: OECD Indicators.

[2] Wengler A, Nimptsch U, Mansky T (2014). Hip and knee replacement in Germany and the USA: analysis of individual inpatient data from German and US hospitals for the years 2005 to 2011. Dtsch Arztebl Int.

[3] Price AJ, Alvand A, Troelsen A, Katz JN, Hooper G, Gray A, Carr A, Beard D (2018). Knee replacement. The Lancet.

[4] Ferguson RJ, Palmer AJ, Taylor A, Porter ML, Malchau H, Glyn-Jones S (2018). Hip replacement. The Lancet.

[5] Müller M, Toussaint R, Kohlmann T (2015). Total hip and knee arthroplasty : Results of outpatient orthopedic rehabilitation [in German]. Orthopade.

[6] Deutsche Rentenversicherung (2011). Reha-Therapiestandards Hüft- und Knie-TEP. Leitlinie für die medizinische Rehabilitation der Rentenversicherung.

[7] Ritter S, Dannenmaier J, Jankowiak S, Kaluscha R, Krischak G (2018). Total Hip and Knee Arthroplasty - Utilization of Postoperative Rehabilitation [in German]. Rehabilitation (Stuttg).

[8] Baulig C, Grams M, Röhrig B, Linck-Eleftheriadis S, Krummenauer F (2015). Clinical outcome and cost effectiveness of inpatient rehabilitation after total hip and knee arthroplasty. A multi-centre cohort benchmarking study between nine rehabilitation departments in Rhineland-Palatinate (Western Germany). Eur J Phys Rehabil Med.

[9] Müller E, Mittag O, Gülich M, Uhlmann A, Jäckel W H (2009). Systematic literature analysis on therapies applied in rehabilitation of hip and knee arthroplasty: methods, results and challenges [in German]. Rehabilitation (Stuttg).

[10] Tuncel T, Simon S, Peters KM (2015). Flexible rehabilitation times after total hip and knee replacement [in German]. Orthopade.

[11] Khan F (2008). Multidisciplinary rehabilitation programmes following joint replacement at the hip and knee in chronic arthropathy. Cochrane Database Syst Rev.

[12] Henderson KG, Wallis JA, Snowdon DA (2018). Active physiotherapy interventions following total knee arthroplasty in the hospital and inpatient rehabilitation settings: a systematic review and meta-analysis. Physiotherapy.

[13] Ottenbacher KJ, Graham JE (2007). The state-of-the-science: access to postacute care rehabilitation services. A review. Arch Phys Med Rehabil.

[14] Bethge M, Mattukat K, Fauser D, Mau W (2017). Rehabilitation access and effectiveness for persons with back pain: the protocol of a cohort study (REHAB-BP, DRKS00011554). BMC Public Health.

[15] Sibold M, Mittag O, Kulick B, Müller E, Opitz U, Jäckel W H (2011). Predictors of participation in medical rehabilitation follow-up in working patients with chronic back pain [in German]. Rehabilitation (Stuttg).

[16] Bini S, Mahajan J (2016). Clinical outcomes of remote asynchronous telerehabilitation are equivalent to traditional therapy following total knee arthroplasty: A randomized control study. J Telemed Telecare.

[17] Moffet Hélène, Tousignant Michel, Nadeau Sylvie, Mérette Chantal, Boissy Patrick, Corriveau Hélène, Marquis François, Cabana François, Ranger Pierre, Belzile Étienne L, Dimentberg Ronald (2015). In-Home Telerehabilitation Compared with Face-to-Face Rehabilitation After Total Knee Arthroplasty: A Noninferiority Randomized Controlled Trial. J Bone Joint Surg Am.

[18] Piqueras M, Marco E, Coll M, Escalada F, Ballester A, Cinca C, Belmonte R, Muniesa J (2013). Effectiveness of an interactive virtual telerehabilitation system in patients after total knee arthoplasty: a randomized controlled trial. J Rehabil Med.

[19] Tousignant M, Moffet H, Boissy P, Corriveau H, Cabana F, Marquis F (2011). A randomized controlled trial of home telerehabilitation for post-knee arthroplasty. J Telemed Telecare.

[20] Pastora-Bernal JM, Martín-Valero Rocio, Barón-López Francisco Javier, Estebanez-Pérez María José (2017). Evidence of Benefit of Telerehabitation After Orthopedic Surgery: A Systematic Review. J Med Internet Res.

[21] Eisermann U, Haase I, Kladny B (2004). Computer-aided multimedia training in orthopedic rehabilitation. Am J Phys Med Rehabil.

[22] Antón D, Nelson M, Russell T, Goñi A, Illarramendi A (2015). Validation of a Kinect-based telerehabilitation system with total hip replacement patients. J Telemed Telecare.

[23] Lauber B, Keller M (2014). Improving motor performance: selected aspects of augmented feedback in exercise and health. Eur J Sport Sci.

[24] Zerpa C (2015). The Use of Microsoft Kinect for Human Movement Analysis. International Journal of Sports Science.

[25] Clark RA, Pua Y, Fortin K, Ritchie C, Webster KE, Denehy L, Bryant AL (2012). Validity of the Microsoft Kinect for assessment of postural control. Gait Posture.

[26] John M MeineReha.

[27] John M (2013). MeineReha® - Gesamtsystem für die Lebensbereich übergreifende Rehabilitatio. e-health 2013 - Informationstechnologien und Telematik im Gesundheitswesen.

[28] Wochatz M, Tilgner N, Mueller S, Rabe S, Eichler S, John M, Völler Heinz, Mayer F (2019). Reliability and validity of the Kinect V2 for the assessment of lower extremity rehabilitation exercises. Gait Posture.

[29] Eichler S, Rabe S, Salzwedel A, Müller Steffen, Stoll J, Tilgner N, John M, Wegscheider K, Mayer F, Völler Heinz, ReMove-It study group (2017). Effectiveness of an interactive telerehabilitation system with home-based exercise training in patients after total hip or knee replacement: study protocol for a multicenter, superiority, no-blinded randomized controlled trial. Trials.

[30] Kennedy DM, Stratford PW, Wessel J, Gollish JD, Penney D (2005). Assessing stability and change of four performance measures: a longitudinal study evaluating outcome following total hip and knee arthroplasty. BMC Musculoskelet Disord.

[31] Salbach NM, O'Brien Kelly K, Brooks D, Irvin E, Martino R, Takhar P, Chan S, Howe J (2015). Reference values for standardized tests of walking speed and distance: a systematic review. Gait Posture.

[32] Dayton MR, Judd DL, Hogan CA, Stevens-Lapsley JE (2016). Performance-Based Versus Self-Reported Outcomes Using the Hip Disability and Osteoarthritis Outcome Score After Total Hip Arthroplasty. American Journal of Physical Medicine & Rehabilitation.

[33] Bade MJ, Struessel T, Dayton M, Foran J, Kim RH, Miner T, Wolfe P, Kohrt WM, Dennis D, Stevens-Lapsley JE (2017). Early High-Intensity Versus Low-Intensity Rehabilitation After Total Knee Arthroplasty: A Randomized Controlled Trial. Arthritis Care Res (Hoboken).

[34] Goldberg A, Chavis M, Watkins J, Wilson T (2013). The five-times-sit-to-stand test: validity, reliability and detectable change in older females. Aging Clin Exp Res.

[35] Bellamy N, Wilson C, Hendrikz J (2011). Population-based normative values for the Western Ontario and McMaster (WOMAC) Osteoarthritis Index: part I. Semin Arthritis Rheum.

[36] van der Wees PJ, Wammes JJG, Akkermans RP, Koetsenruijter J, Westert GP, van Kampen A, Hannink G, de Waal-Malefijt M, Schreurs BW (2017). Patient-reported health outcomes after total hip and knee surgery in a Dutch University Hospital Setting: results of twenty years clinical registry. BMC Musculoskelet Disord.

[37] Quintana J, Escobar A, Bilbao A, Arostegui I, Lafuente I, Vidaurreta I (2005). Responsiveness and clinically important differences for the WOMAC and SF-36 after hip joint replacement. Osteoarthritis Cartilage.

[38] Kennedy DM, Stratford PW, Hanna SE, Wessel J, Gollish JD (2006). Modeling early recovery of physical function following hip and knee arthroplasty. BMC Musculoskelet Disord.

[39] Beck H, Beyer Franziska, Gering Franziska, Günther Klaus-Peter, Lützner Cornelia, Walther Achim, Stiehler Maik (2019). Sports Therapy Interventions Following Total Hip Replacement. Dtsch Arztebl Int.

[40] Ellert U, Kurth B (2004). Methodological views on the SF-36 summary scores based on the adult German population [in German]. Bundesgesundheitsblatt Gesundheitsforschung Gesundheitsschutz.

[41] Bundesagentur für Arbeit (2017). Akademikerinnen und Akademiker.

[42] Bundesagentur für Arbeit (2018). Statistik nach Regionen: Berlin.

[43] Egger M, Juni P, Bartlett C, Holenstein F, Sterne J (2003). How important are comprehensive literature searches and the assessment of trial quality in systematic reviews? Empirical study. Health Technol Assess.

