# Supplementary material for: The Effectiveness of Telerehabilitation as a Supplement to Rehabilitation in Patients After Total Knee or Hip Replacement: Randomized Controlled Trial
Source: JMIR Rehabil Assist Technol. 2019 Nov 7;6(2):e14236. doi: 10.2196/14236 (PMC6873150; doi:10.2196/14236)
Supplement: Multimedia Appendix 1 [file rehab_v6i2e14236_app1.pdf]

CONSORT-EHEALTH Checklist V1.6.2 Report	Manuscript Number	14236
**(based on CONSORT-EHEALTH V1.6), available at [http://tinyurl.com/consort-ehealth-v1-6].**		
**Date completed**		
4/2/2019 6:22:46		
**by**		
Sarah Eichler		
Telerehabilitation as a supplement to usual aftercare in patients after total knee or hip replacement - A randomized controlled study for the effectiveness of a telemedical assisted movement therapy in orthopaedic rehabilitation		
TITLE		
**1a-i) Identify the mode of delivery in the title**		
Telerehabilitation		
**1a-ii) Non-web-based components or important co-interventions in title**		
**1a-iii) Primary condition or target group in the title**		
patients after total knee or hip replacement		
ABSTRACT		
**1b-i) Key features/functionalities/components of the intervention and comparator in the METHODS section of the ABSTRACT**		
Telerehabiliation		
**1b-ii) Level of human involvement in the METHODS section of the ABSTRACT**		
patients after total knee or hip replacement		
**1b-iii) Open vs. closed, web-based (self-assessment) vs. face-to-face assessments in the METHODS section of the ABSTRACT**		
rehab center		
**1b-iv) RESULTS section in abstract must contain use data**		
111		
**1b-v) CONCLUSIONS/DISCUSSION in abstract for negative trials**		
equivalent to usual aftercare		
INTRODUCTION		
**2a-i) Problem and the type of system/solution**		
The aim of this randomized controlled trial was to examine the previously developed telemedical assisted movement therapy regarding functional parameters, quality of life and pain as well as return to work compared to usual aftercare.		
**2a-ii) Scientific background, rationale: What is known about the (type of) system**		
In this regard, telerehabilitation seems predestined since it can be performed regardless of location and time. Therefore, it has the potential to increase both utilization and therapy adherence. The telemedical offers should be adapted to the individual and indication specific needs of the patients and should enable contact with the supervising therapists. However, this could not be adequately investigated with the systems available so far[14-20] as they are either not specific enough to the indication or do not have the opportunity to communicate with a therapist. The system MeineReha® [21, 22] combines these components and also provides real-time visual feedback. After development and validation, the system MeineReha® was supplemented with an individualized and therapist controlled telemedical assisted movement therapy consisting of 38 available training exercises for patients after knee as well as after hip replacement.		
**Does your paper address CONSORT subitem 2b?**		

The telemedical offers should be adapted to the individual and indication specific needs of the patients and should enable contact with the supervising therapists. However, this could not be adequately investigated with the systems available so far.		
METHODS		
**3a) CONSORT: Description of trial design (such as parallel, factorial) including allocation ratio**		
were included in the randomized controlled trial		
**3b) CONSORT: Important changes to methods after trial commencement (such as eligibility criteria), with reasons**		
not applicable, no changes		
**3b-i) Bug fixes, Downtimes, Content Changes**		
**4a) CONSORT: Eligibility criteria for participants**		
Patients were eligible for inclusion if a total hip or knee replacement was done after idiopathic, posttraumatic or congenital osteoarthritis, they were aged between 18 and 65 years and insured by the national or regional German Pension Insurance.		
**4a-i) Computer / Internet literacy**		
**4a-ii) Open vs. closed, web-based vs. face-to-face assessments:**		
in three inpatient rehabilitation centers		
**4a-iii) Information giving during recruitment**		
**4b) CONSORT: Settings and locations where the data were collected**		
in three inpatient rehabilitation centers, study site University of Potsdam		
**4b-i) Report if outcomes were (self-)assessed through online questionnaires**		
not applicable		
**4b-ii) Report how institutional affiliations are displayed**		
rehab centers, University of potsdam		
**5) CONSORT: Describe the interventions for each group with sufficient details to allow replication, including how and when they were actually administered**		
**5-i) Mention names, credential, affiliations of the developers, sponsors, and owners**		
**5-ii) Describe the history/development process**		
**5-iii) Revisions and updating**		
**5-iv) Quality assurance methods**		
**5-v) Ensure replicability by publishing the source code, and/or providing screenshots/screen-capture video, and/or providing flowcharts of the algorithms used**		
**5-vi) Digital preservation**		
**5-vii) Access**		
randomized		
**5-viii) Mode of delivery, features/functionalities/components of the intervention and comparator, and the theoretical framework**		

individual training therapy, set up by physiotherapists		
**5-ix) Describe use parameters**		
three times a week		
**5-x) Clarify the level of human involvement**		
technical support, University of Potsdam		
**5-xi) Report any prompts/reminders used**		
messages from the physiotherapists		
**5-xii) Describe any co-interventions (incl. training/support)**		
voluntary use of usual aftercare		
**6a) CONSORT: Completely defined pre-specified primary and secondary outcome measures, including how and when they were assessed**		
primary endpoint (improvement in the 6-minute walk test)		
**6a-i) Online questionnaires: describe if they were validated for online use and apply CHERRIES items to describe how the questionnaires were designed/deployed**		
**6a-ii) Describe whether and how "use" (including intensity of use/dosage) was defined/measured/monitored**		
**6a-iii) Describe whether, how, and when qualitative feedback from participants was obtained**		
**6b) CONSORT: Any changes to trial outcomes after the trial commenced, with reasons**		
in three inpatient rehabilitation centers, study site University of Potsdam		
**7a) CONSORT: How sample size was determined**		
**7a-i) Describe whether and how expected attrition was taken into account when calculating the sample size**		
see published study protocol		
**7b) CONSORT: When applicable, explanation of any interim analyses and stopping guidelines**		
primary endpoint (improvement in the 6-minute walk test)		
**8a) CONSORT: Method used to generate the random allocation sequence**		
using block randomization in the ratio of 1: 1, based on randomization lists drawn up in advance by the biometric institute. Written consent was obtained from all patients.		
**8b) CONSORT: Type of randomisation; details of any restriction (such as blocking and block size)**		
using block randomization in the ratio of 1: 1, based on randomization lists drawn up in advance by the biometric institute. Written consent was obtained from all patients.		
**9) CONSORT: Mechanism used to implement the random allocation sequence (such as sequentially numbered containers), describing any steps taken to conceal the sequence until interventions were assigned**		
not applicable		
**10) CONSORT: Who generated the random allocation sequence, who enrolled participants, and who assigned participants to interventions**		
biometrical institute		
**11a) CONSORT: Blinding - If done, who was blinded after assignment to interventions (for example, participants, care providers, those assessing outcomes) and how**		
**11a-i) Specify who was blinded, and who wasn't**		
Blinding of the patients was not possible dur to the nature of the trial...Another limitation of the study design is the lack of blinding of study participants and investigators. As a result, a possible influence on the participants during the investigations cannot be excluded.		
**11a-ii) Discuss e.g., whether participants knew which intervention was the "intervention of interest" and which one was the "comparator"**		

**11b) CONSORT: If relevant, description of the similarity of interventions**		
not applicable		
**12a) CONSORT: Statistical methods used to compare groups for primary and secondary outcomes**		
The statistical analyzes were conducted according to the description in the previously published study protocol.[23] All analyzes were performed with the full analysis set of randomized patients (intention-to-treat). Patient characteristics and follow-up values were described descriptively with mean and standard deviation (metric variables) and absolute and relative frequencies (categorical variables). Group-specific changes in metric variables (trends) were tested for significance versus "no change" with one-factorial variance analyzes. The calculation of the number of cases (n = 84) was based on the comparison of the primary endpoint (improvement in the 6-minute walk test) between the groups. This comparison was carried out with an analysis of covariance (ANCOVA) with 22 baseline covariates at the 5 % level (two-sided). All metric secondary endpoints were tested analogously without multiple adjustment. The ANCOVA estimates of the group differences in the continuous endpoints are presented in a forest plot. The group difference of the return-to-work rate was tested with the Chi2-test.		
**12a-i) Imputation techniques to deal with attrition / missing values**		
see above		
**12b) CONSORT: Methods for additional analyses, such as subgroup analyses and adjusted analyses**		
not applicable		
RESULTS		
**13a) CONSORT: For each group, the numbers of participants who were randomly assigned, received intended treatment, and were analysed for the primary outcome**		
At baseline, data of 92 patients (IG: n = 48, CG: n = 44, Figure 1) could be collected.		
**13b) CONSORT: For each group, losses and exclusions after randomisation, together with reasons**		
Figure 1		
**13b-i) Attrition diagram**		
**14a) CONSORT: Dates defining the periods of recruitment and follow-up**		
From August 2016 to December 2017,		
**14a-i) Indicate if critical “secular events” fell into the study period**		
**14b) CONSORT: Why the trial ended or was stopped (early)**		
not applicable		
**15) CONSORT: A table showing baseline demographic and clinical characteristics for each group**		
Table 1		
**15-i) Report demographics associated with digital divide issues**		
Table 1		
**16a) CONSORT: For each group, number of participants (denominator) included in each analysis and whether the analysis was by original assigned groups**		
**16-i) Report multiple “denominators” and provide definitions**		
At baseline, data of 92 patients (IG: n = 48, CG: n = 44, Figure 1) could be collected.		
**16-ii) Primary analysis should be intent-to-treat**		
**17a) CONSORT: For each primary and secondary outcome, results for each group, and the estimated effect size and its precision (such as 95% confidence interval)**		
Table 2		
**17a-i) Presentation of process outcomes such as metrics of use and intensity of use**		

**17b) CONSORT: For binary outcomes, presentation of both absolute and relative effect sizes is recommended**		
Table 2		
**18) CONSORT: Results of any other analyses performed, including subgroup analyses and adjusted analyses, distinguishing pre-specified from exploratory**		
not applicable		
**18-i) Subgroup analysis of comparing only users**		
**19) CONSORT: All important harms or unintended effects in each group**		
not applicable		
**19-i) Include privacy breaches, technical problems**		
**19-ii) Include qualitative feedback from participants or observations from staff/researchers**		
DISCUSSION		
**20) CONSORT: Trial limitations, addressing sources of potential bias, imprecision, multiplicity of analyses**		
**20-i) Typical limitations in ehealth trials**		
In the investigated sample, an above-average education level can be ascertained (43.5 % with polytechnic or university degree). Data from the Employment Agency in Germany show that in the total population only 20 % of the gainfully employed persons have a polytechnic or university degree.[35] Furthermore, the unemployment rate of the sample with a rate of 5.4 % is to be classified as low compared to the average in Berlin of 8.4 %.[36] In addition, a large proportion of the patients came from Berlin and the surrounding countryside. Therefore, in this study, the access route to the study site the patients had to do independently and twice may have been an obstacle to the participation of patients from structurally weak areas far off. Only a quarter of the screened patients participated in the study. Thus, the low participation rate and the discussed patient characteristics suggest a selection bias.<br>Another limitation of the study design is the lack of blinding of study participants and investigators. As a result, a possible influence on the participants during the investigations cannot be excluded. It is known that in non-blinded studies greater intervention effects than in blinded ones can be shown.[37]<br>All patients underwent inpatient rehabilitation and aftercare. It is not possible to determine which improvements can be directly traced back to the effect of telerehabilitation, as due to ethical reasons usual aftercare in this study were not replaced but were complementarily extended.		
**21) CONSORT: Generalisability (external validity, applicability) of the trial findings**		
**21-i) Generalizability to other populations**		
**21-ii) Discuss if there were elements in the RCT that would be different in a routine application setting**		
**22) CONSORT: Interpretation consistent with results, balancing benefits and harms, and considering other relevant evidence**		
**22-i) Restate study questions and summarize the answers suggested by the data, starting with primary outcomes and process outcomes (use)**		
The investigated telemedical assisted exercise therapy in patients after knee or hip replacement was equivalent to the usual aftercare regarding the difference achieved in the 6-minute walk test. In addition, equivalent increases in both groups were demonstrated as secondary endpoints for functional mobility, health-related quality of life and joint-related complaints. However, the patients of the intervention group were employed at a significantly higher rate at the end of the intervention.		
**22-ii) Highlight unanswered new questions, suggest future research**		
Other information		
**23) CONSORT: Registration number and name of trial registry**		
German Register of Clinical Trials (ID DRKS00010009).		

**24) CONSORT: Where the full trial protocol can be accessed, if available**		
Eichler, S., et al., Effectiveness of an interactive telerehabilitation system with home-based exercise training in patients after total hip or knee replacement: study protocol for a multicenter, superiority, no-blinded randomized controlled trial. Trials, 2017. 18(1): p. 438		
**25) CONSORT: Sources of funding and other support (such as supply of drugs), role of funders**		
The study was funded by the German Pension Insurance Berlin-Brandenburg (grant number 10-40.07.05.07.007).		
**X26-i) Comment on ethics committee approval**		
The study protocol with the description of the methods was written and published in accordance with the ethical requirements of the current version of the Declaration of Helsinki (Revision 2013).[23] The Ethics Committee of the University of Potsdam gave a positive ethics vote (No. 15/2016).		
**x26-ii) Outline informed consent procedures**		
Written consent was obtained from all patients.		
**X26-iii) Safety and security procedures**		
**X27-i) State the relation of the study team towards the system being evaluated**		
The Authors declare no Competing Financial or Non-Financial Interests.		
